# Worldwide Forensic Dermatology

**DOI:** 10.7759/cureus.84645

**Published:** 2025-05-22

**Authors:** Philip R Cohen

**Affiliations:** 1 Dermatology, University of California Davis School of Medicine, Sacramento, USA; 2 Dermatology, Touro University California College of Osteopathic Medicine, Vallejo, USA; 3 Maples Center for Forensic Medicine, University of Florida College of Medicine, Gainesville, USA

**Keywords:** cutaneous, dermatology, education, forensic, hair, medicine, mucosa, nails, pathology, skin

## Abstract

Forensic medicine can involve the skin, mucosa, hair, and nails. Traditionally limited to examinations of decedents, forensic medicine now also encompasses age estimation, civil and criminal case investigations, and assessments of live victims of torture, human trafficking, and traffic accidents. Forensic dermatology, a recently emerging field in India, focuses on determining causes of death by distinguishing drug reactions from traumatic injuries and assessing the injuries in victims of human trafficking, torture, assault, deprivation, neglect, and abuse. Core elements of all forensic disciplines, including forensic dermatology, are education, documentation, and collaboration. Currently, the training of medical students and practitioners in forensic dermatology is insufficient. This educational gap can be addressed by incorporating forensic pathology and dermatology into the first two years of medical school curricula. The national dermatology association in each country can enhance it by requiring forensic dermatology training during dermatology residency, creating a dedicated forensic dermatology task force, and integrating forensic dermatology seminars into national dermatology conferences. Effective communication of documented findings is another essential component of forensic dermatology. As with other forensic specialties (such as anthropology, ballistics, entomology, and odontology), expert analytical reports are essential for conveying relevant findings. A standardized template for forensic dermatology expert analytical reports has recently been developed. Interdisciplinary collaboration and research are also vital to the advancement of forensic dermatology. A notable example is the development of a practical, non-racial, and non-ethnic colorimetric scale for skin of color, created through the collaboration of a dermatologist and three forensic pathologists. This scale is already in use by forensic pathologists in their evaluation of decedents with various skin tones. In conclusion, forensic dermatology is essential for the medical evaluation and criminal investigation of both decedents and certain live victims. Dermatologists are uniquely positioned to advance the field through education, practical application, and continued research.

## Editorial

Forensic dermatology has recently been unveiled in India [1]. When skin-related evidence is found at a crime scene, the expertise of dermatologists should be incorporated in the criminal investigation. Currently, there is a lack of forensic training in the dermatology curriculum in medical schools, as well as limited integration of forensic evaluation into clinical dermatology practice. Three key components of forensic dermatology are education, documentation, and collaboration.

Traditionally, forensic medicine has been limited to the evaluation of decedents. However, in many countries, physicians are now also involved in assessing live victims, not only of traffic accidents but also of torture. Dermatologists are uniquely positioned to examine the skin, mucous membranes, hair, and nails of both the living and the deceased. Indeed, forensic dermatology holds the potential to identify victims of human trafficking and to distinguish drug reactions that may mimic traumatic injuries [2,3].

Training in forensic dermatology should begin in the medical school. In most medical schools, the first two years of classes include a substantial number of lectures on general pathology and a much less extensive exposure to dermatology. This imbalance could be addressed by incorporating lectures on both forensic pathology and forensic dermatology into the early medical curriculum [1].

Currently, most dermatology residency programs do not include dedicated instruction or electives in forensic dermatology. National dermatology associations in each country should advocate for the integration of forensic dermatology into residency training. Structured education in this field should be an integral part of the residency curriculum. In addition, national dermatology associations should elevate forensic dermatology as a key area of focus by establishing dedicated task forces and offering relevant, instructive seminars at their conferences.

Uniform documentation of forensic dermatology findings is crucial. The development of standardized guidelines for documenting observations and handling evidence, which could serve as reference documents for all dermatologists, is essential. In other forensic specialties (such as anthropology, ballistics, entomology, and odontology), specialists routinely prepare a forensic expert analytical report. A template for succinctly presenting findings related to the skin, mucosa, hair, and nails of a decedent in a forensic dermatology expert analytical report has recently been published [4].

To enhance the utilization of forensic dermatology, fostering interdisciplinary collaboration and research is imperative. Indeed, the joint efforts of a dermatologist and three forensic pathologists led to the development of a practical and easy-to-use colorimetric scale for skin of color, which is neither based on race nor ethnicity of the person [5]. This scale has already been adopted by forensic pathologists in their evaluation of decedents with skin of color [5].

Forensic dermatology represents the newest frontier in forensic medicine. By initiating instrumental changes in education, documentation, and collaboration, dermatologists are in an excellent position to promote the training, utilization, and investigation in this area, which encompasses both medical evaluation and criminal investigation. In conclusion, forensic dermatology is an essential and growing component of global forensic practice.

## References

[1] James RI, Sathishkumar D (2025). Contours of justice: unveiling forensic dermatology in India. Indian Dermatol Online J.

[2] Cohen PR (2023). Identifying human trafficking victims: a potential role for forensic dermatology. Cureus.

[3] Cohen PR (2023). The cutaneous manifestations of drug reactions can mimic traumatic injuries: case reports and the potential role of forensic dermatology. Cureus.

[4] Cohen PR, Sutton L (2025). Forensic dermatology expert analytical report: a new frontier of forensic medicine. Dermatol Ther (Heidelb).

[5] Cohen PR, DiMarco MA, Geller RL, Darrisaw LA (2023). Colorimetric scale for skin of color: a practical classification scale for the clinical assessment, dermatology management, and forensic evaluation of individuals with skin of color. Cureus.

